# A pilot study investigating the relationship between heart rate variability and blood pressure in young adults at risk for cardiovascular disease

**DOI:** 10.1186/s40885-021-00185-z

**Published:** 2022-01-15

**Authors:** Linda P. Bolin, Amelia D. Saul, Lauren L. Bethune Scroggs, Carolyn Horne

**Affiliations:** 1 College of Nursing, East Carolina University, Greenville, United States; 2 Department of Addiction and Rehabilitation Studies, East Carolina University, Greenville, United States

**Keywords:** Biofeedback, Breathing exercises, Blood pressure, Young adult

## Abstract

**Background:**

Cardiovascular disease is one of the leading causes of death globally with hypertension being a primary cause of premature death from this disease process. Individuals with a family history of cardiovascular disease and hypertension are at a greater risk for developing the same sequela. Autonomic cardiac control is important in the level of cardiac function. One intervention that is effective in improving cardiovascular function is heart rate variability biofeedback training. The purpose of our study was to determine the effectiveness of heart rate biofeedback training on HRV and blood pressure in individuals with a family history of cardiovascular disease.

**Methods:**

Thirty-four participants (76.5% female, 22.7 ± 4.3 years) completed a baseline assessment and training using an established short-term HRV protocol followed by two weeks of at-home paced breathing employing a smartphone application. The participants were then reassessed in a biofeedback clinic.

**Results:**

The participants physiological measures showed a significant increase in means between pre and post intervention of SDNN *(t* (32) = 2.177, *p* =.037) and TP, (*t* (32) = 2.327 *p* = .026). Correlation noted a medium effect on diastolic blood pressure and high frequency heart rate variability, F, *r* = .41, *n* =33, *p* < .05. A multiple regression with all predictor variables in the model found no significance with diastolic and systolic blood pressure.

**Conclusions:**

The findings from this pilot study demonstrated that a two-week paced breathing intervention may assist in reducing heart rate and diastolic blood pressure while improving heart rate variability.

**Supplementary Information:**

The online version contains supplementary material available at 10.1186/s40885-021-00185-z.

## Background

High blood pressure (BP), also referred to as hypertension (HTN), accounts for the second-largest number of preventable cardiovascular (CV) deaths. Globally there are an estimated 1.13 billion people with diagnosed HTN with approximately 20% having their HTN under control. [1] The American Heart Association Hypertension Clinical Guidelines changed in 2017, lowering BP readings in Stage I to 130/80. It is estimated that this will result in a 14% increase of those with a new diagnosis of HTN, and it is expected to be greatest among younger adults. [2] However, these guidelines were only adopted in the U.S and not globally. [3–5].

Given current U.S. guidelines, the diagnosis of HTN may occur at an earlier age resulting in a longer lifetime burden of the condition. Therefore, slowing the onset or progression of HTN can substantially reduce cardiovascular disease.[6, 7] Multiple studies over time confirm that reducing BP reduces mortality. [8–10] Seminal studies include the Framingham Heart Study [11], which explored BP in normotensive and untreated individuals with hypertension over 30 years, and the Veterans Administration Cooperative Study, [12] which emphasized the need for early systematic efforts to screen those at risk for developing HTN due to the asymptomatic nature of the disease. Most recently, the Systolic Blood Pressure Intervention Trial (SPRINT) demonstrated that targeting a systolic blood pressure (SBP) of less than 120 mmHg resulted in lower rates of fatal and nonfatal major cardiovascular events and death from any cause. [13].

Many of these studies focused on medication management once individuals were diagnosed with HTN. For those at risk, the main focus is on modifiable risk factors such as decreasing dietary salt intake, increasing physical activity, and dietary modification. Although progress has been made in the prevention of HTN, it continues to be a major public health challenge for those at-risk, warranting the need to explore new and early self-management interventions.

BP regulation is influenced by the autonomic nervous system (ANS). The sympathetic nervous system can increase heart rate and raise BP. Heart rate variability (HRV) is a physiological measure of the change in variability between successive heartbeats and reflects ANS function. HRV is measured in milliseconds (ms) during the R-R interval, sometimes referred to as the inter-beat interval (IBI). Past studies have shown that impairments of the ANS contribute to the development of cardiovascular disease and HTN. [14–16] According to Shaffer and Ginsberg [17], HRV is optimal when associated with self-regulation, adaptability, and resilience. A low HRV is an indication of the body being under stress and a marker for the possibility of cardiac death. [16, 18] Acute episodes of anxiety or stress trigger physiological responses that increase cardiac output and heart rate, resulting in an increased BP. Prolonged stress over time results in vascular hypertrophy or atherosclerosis, lending to HTN and cardiovascular disease (CVD). [19–21] Furthermore, when modifiable and unmodifiable, such as family history, risk factors are compounded in hypertensive patients, and heart rate variability is decreased. [22].

Gaining an understanding of the ANS and practicing techniques to regulate bodily functions can achieve a better HRV and prove beneficial in BP regulation. An intervention used for autonomic strengthening is paced breathing, a biofeedback (BF) technique to manage HRV. [23–25] Slow, paced breathing at short intervals can control the fight-flight response, causing vasodilation and a decrease in BP. Baroreceptors located in the carotid arteries and aortic arches regulate the ANS. Inhalation causes an increase in heart rate, whereas exhalation decreases heart rate followed by decreased BP within 5 s.[26, 27] Lin and colleagues [28] recently demonstrated that mobile applications (apps) could effectively increase cardiac autonomic balance. For our study, a repeated measure design was used to explore differences in heart rate variability of young adults with a family history of cardiovascular disease before and after a paced breathing intervention. Purposes of the study were to explore how SBP and DBP alone correlate with time and frequency of HRV and if there is an effect on BP after training in paced breathing in a young population that has not been diagnosed with HTN.

## Methods

### Participants

Approval was obtained from the University and Medical Center Institutional Review Board (UMCIRB). The sample consisted of young adults with the inclusion criteria: (1) between 18 and 35 years of age, (2) family history of CVD, and (3) English speaking. Exclusion criteria consisted of (1) documented diagnosis of HTN, (2) currently prescribed antihypertensive medication, and (3) cognitive impairments that inhibit understanding of instructions. Recruitment flyers were posted in public locations. Those interested contacted the research team and were scheduled for an appointment to determine eligibility. Appointments were scheduled in a BF clinic setting where the study was explained in detail and informed consent was obtained on eligible subjects. A convenience sample of adults (*N* = 34) was recruited.

### Questionnaires

Investigator tools were developed to obtain participants’ demographics, health assessment data, and CV family history. Demographic intake data included: age, sex, ethnicity, marital status, educational level, occupation, and current employment status. Health assessment data included: current medical conditions, current medication, co-existing medical conditions, prescription medications, over-the-counter medications, exercise frequency, intensity, duration, dietary preferences, smoking history, and a self-rating of overall health.

Family history intake included identification of family members with CVD, the specific disease(s) including myocardial infarction, hypertension, stroke, hyperlipidemia, diabetes mellitus, CV related surgeries, overall general health of immediate family members, if family members were alive or deceased and if deceased at what age. All tools were administered via the Research Electronic Data Capture (REDCap) system.

### HRV Indices

Measurements of HRV were obtained using an ear-clip photoplethysmography and software (Heart Tracker, Biocom Technologies, USA) for analyses. The standard of HRV historically relied on electrocardiogram readings. However, with advanced technology, photoplethysmography has shown equivalence in measurement for short-term HRV readings. [29] For this short-term HRV study of young adults who were normatively healthy, photoplethysmography was appropriate for the measurement of HRV parameters.

Low frequency (LF), high frequency (HF), and very low frequency (VLF) are different frequencies with varying power hertz ranges. [17] These ranges equate to measurements that are expressed in milliseconds squared (ms^2^). LF can reflect sympathetic, parasympathetic activity, and blood pressure regulation by baroreceptor activity at rest. With a paced slow breathing exercise, the baroreceptor response may increase LF activity. HF is synchronous with respiratory efforts and reflects parasympathetic action. Causes of low HF may include stress and anxiety. VLF is associated with health. Abnormalities in this frequency may be associated with mortality. The ratio of LF to HF is expressed as LF/HF and is used to quantify the degree of sympathetic and parasympathetic balance in the body. LF/HF is also expressed in ms^2^. If the LF/HF ratio is low then parasympathetic action is dominant, while if high, it reflects sympathetic action. Total power (TP) is a cumulative reflection of the HF, LF, VLF and ultra-low frequency spectral bands and reflects how much power the body has in its ability to adapt. A decrease in this value will typically be seen when the body is under stress. [17].

The time domains measured in this study included root mean squared (RMS) and the standard deviation of N to N (SDNN). The RMS time domain related to HRV is the mathematical calculation resulting in the beat-to-beat variance in heart rate and reported as milliseconds (ms). HF domain correlates with RMS. SDNN is the standard deviation of normal sinus beat-to-beat variances measured in ms. Both sympathetic and parasympathetic activity contribute to SDNN measurement with it being highly correlated with the LF and VLF frequency bands.

Other biometrics included noninvasive continuous BP measurements using the Continuous Non-Invasive Arterial Pressure (CNAP) system with a slip-on design finger sensor (CNSystem®, Austria). The CNAP system is one of a few devices currently available for noninvasive continuous BP measurement. The CNAP is most commonly used during intraoperative procedures as it allows for uninterrupted recordings over long durations. CNAP devices, using an inflatable finger cuff or FINger Arterial PRESsure (Finapres) system, measure arterial pressure based on the principle of dynamic vascular unloading in the arterial walls within the finger. Finapres systems were originally developed in the 1980 s to provide reliable continuous blood pressure monitoring and have proven a reliable alternative for invasive measurements for mean and diastolic pressures [30]. Imholz and colleagues [31] reviewed Finapres technology and concluded the accuracy and precision are sufficient for tracking BP changes. Though recent research has contended that CNAP systems are inaccurate for patient care decision making, a CNAP system was used in this study for its ability to noninvasively and continuously monitor patient blood pressure in the research setting. [32] Additionally, with each participant, an initial BP measurement was obtained using a traditional BP cuff and these measures were correlated with the initial CNAP measurement. A clear advantage of using continuous BP measurement is the change is noted instantly with intervention, such as paced breathing. However, it is not advantageous to use at home due to equipment complexity since was developed to be used in healthcare settings.

### Mobile applications

Home training tools consisted of a digital application (app) for paced breathing; Breath+ (iPhone) and Breathe2Relax (Androids). Although no research has been done crossing two applications, both are designed to allow users to set breathing pace rates depending on which type of mobile phone they possess. For our study, these applications were used primarily to reinforcement the paced breathing taught in the BF clinic during the initial session. The two free applications were provided to participants for their at-home training because both are designed so that users can set a paced breathing rate. These specific mobile applications have a similar user interface to the Heart Tacker software used during the initial face to face training session at the BF clinic. The applications allowed participants with either operating system (apple or android) to have access to a mobile paced breathing application to continue training at home. There are minimal differences between these applications.

In this pilot study, participants were seen in our BF clinic, a private, temperature-controlled room with no ambient noise. The room’s physical design remained consistent throughout the study with the same BF chair, BF equipment, software for collecting heart rate variability data, paced breathing training software, and a continuous blood pressure monitoring system. As we assessed the practicality of our study and wanted to assure participant retention, a 3-session design, with each session lasting 1 to 1 ½ hours, was agreed upon. A short-term HRV protocol was utilized. According to Voss and colleagues [33], short-term HRV is acceptable and has the benefits of providing immediate results in settings or with participants with time restraints. An individual’s heart rate and breathing synchronize at a specific breathing rate; this synchronization is called their resonance frequency. Each person has a unique resonance frequency related to their breathing rate, and breathing at a rate outside of this frequency can cause stress and impact HRV measures. [34] Many studies have found maximum effects while participants are breathe at approximately six breaths per minute or 0.1 Hz though there is evidence that resonance can occur at lower frequencies. [35].

The first session provided an intake of baseline data and pre-testing. The second session involved a training session with detailed home instruction for continuing training. The third session consisted of post-testing. All sessions were documented and completed by trained BF providers.

Before each session, participants were instructed not to consume caffeinated beverages within 3 h, not to eat a heavy meal within two hours, not to engage in aerobic exercise for at least one hour, and not to smoke within 30 min before the session. Participants were also asked to bring their mobile devices to each appointment.

At the baseline session, participants were placed in the quiet clinic room to complete all intake questionnaires. A brief description followed, describing the equipment, purpose, and procedures. Equipment was connected to the participant, who was asked to sit quietly for five minutes with legs uncrossed, and feet flat and comfortably on the floor. Baseline BP measurements were obtained, and a comparison was made between arm cuff pressures and finger cuff pressures for validation. Afterwards, skin preparation for BP monitoring and HRV sensor application was completed. BF sensors were connected, and with the participant breathing normally, baseline measurements for BP and HRV were obtained for five minutes. For consistency, in our protocol, all participants completed paced breathing at a rate of 5.5 to 6 breaths per minute, using guidance by an established HRV protocol. [36].

The second session included giving the participant thorough instructions on breathing using a paced breathing computer software application. The reinforcement of the training session was enhanced with a visual screen depicting each participant’s progress and compliance. Prior to the paced breathing training, a baseline BP reading was obtained. During the five-minute paced breathing cycle, BPs were obtained every minute. Participants were observed for any respiratory compromise. Signs of hyperventilation, including lightheadedness, dizziness, or an increase in heart rate were monitored. All data were recorded from the baseline and training sessions.

After the in-clinic session was completed, participants were assisted in loading the breathing app on their mobile devices. Two breathing apps were used with the breath settings standard across both devices to accommodate both iPhone and Android users. Theses apps modeled the same paced breathing as the software program used in the BF clinic setting. Participants were instructed to breathe at the resonant frequency of 5.5 to 6 breaths per minute, twice a day for 10 min each at-home session until returning for their next visit to the BF clinic.

The final session followed the same procedure as the second session for consistency in collecting and comparing data. In addition, participant intake also included the frequency and length of their mobile app training sessions. Again, participants were observed for any signs of hyperventilation. Participants were given visual copies of their progress from baseline to session 3, along with a detailed explanation, and then thanked for their participation. Participants were also encouraged to continue using the app for self-management purposes as needed.

### Data analyses

Descriptive statistics were used for sample description. Independent t-tests were used for the continuous variables of heart rate (HR), SBP, DBP and, HRV measures at baseline and after training. Correlation using Pearson-r was used to determine the strength and direction of variable relationships. Multiple regression was used to determine the variance of HRV on both SBP and DBP. All data were analyzed using Statistical Package for the Social Sciences (SPSS), version 26.0.

## Results

Participants were primarily female (76.5%) and White (79.4%) with a mean age of 22.7 ± 4.3 years. The majority reported overall excellent to good health (88%), with the remainder being fair or below. Anxiety was reported among 38% of the participants as being a problem. Most reported no history of having any high BP readings in the past (91%). Fatigue-related to sleep was an issue in 29% of participants. Family medical history included hypertension (91%), high cholesterol (76%), diabetes (47%), and previous heart operation (41%). See Table 1 for demographics.

**Table 1:** Demographics (*N* = 34)

Variable	Frequency (***n***)
Sex	
Female	76.5% (26)
Male	23.5% (8)
Race	
Black	14.7% (5)
White	79.4% (27)
Other	5.9% (2)
Marital Status*	
Married	17.6% (6)
Single	79.4% (27)
Education	
High School	35.3% (12)
College	61.8% (21)
Graduate School	21.9% (1)
Self-Rated Health	
Excellent	20.6% (7)
Good	67.6% (23)
Fair	11.8% (4)
Participant Reported Problems	
Heart Racing	32.4% (11)
Extra Heart Beats	20.6% (7)
Anxiety	38.2% (13)
Fatigue	29.4% (10)
Family Health History	
Myocardial Infarction	14.7% (5)
Hypertension	91.2% (31)
Hypercholesteremia	76.5% (26)
Diabetes	47.1% (16)
Heart Operation	41.2% (14)

*one participant did not provide marital status

The baseline mean HR for the sample was 82 ± 11 beats per minute (bpm). The baseline SBP was 119 ± 16 mmHg. while the mean DBP was 75 ± 14 mmHg. Minimum SDNN at baseline was 21.7 ms with a maximum of 104.5 ms (*M* = 52.32 ± 22.87 ms).

Paired sample t-tests were completed for HR, SBP, DBP, LF HF, very low frequency (VLF), LF/HF, SDNN and TP. No significance was found in HR from baseline (*M* = 81.71 ± 11.03 bpm) to after HRV training (*M*= 81.58 ± 13.41 bpm), *t* (32) = 0.07, *p* =.945. SBP showed an increase in mean from baseline (*M* = 121.33 ± 15.46 mmHg) to after training (*M* = 122 ± 18.10 mmHg), *t* (32) = 1.27, *p* =.63. DBP was close to significance when comparing means, (*M* = 74.79 ± 11.04 mmHg) to after training (*M* = 73.09 ± 0.24 mmHg), *t* (32) = 1.93, *p* = .06. However, there was an increase in SDNN showing a significance when comparing the means before (*M* = 52.77 ± 4.02 ms) to after training (*M* = 63.27 ± 29.29 ms), *t* (32) = 2.177, *p* =.037. TP showed an increase with significance (*M* = 955.42 ± 858.34 ms) to after training (*M* = 1528.1 ± 1467.80 ms), *t* (32) = 2.327, *p* = .026. LF also showed increased significance after training (*M*=5.44 ± 1.01 ms), *t*(32) = -1.99, *p* = .05. LF also showed increased significance from before training (*M*=5.44 ± 1.01 ms) to after training (*M* =5.861 ± 1.36, *t*(32) = -1.99, *p* = .05. No significance was found with HF, VLF or LF/HF. Eta square values for all t-tests had small effect sizes.

Pearson’s product correlation was used to explore the relationships with variables and their direction. SBP did not show any correlation with HRV time and frequency variables. However, DBP did show a significance (*p* <.05, 2-tailed) with HF. There was a medium, negative correlation between these variables, *r* = .41, *n* =33, *p* < .05. No other correlational significance was found between BP and HRV variables. See Table 2.

**Table 2:** Pearson Product-moment Correlations Between BP and HRV (*n*=33)

Variable	1	2	3	4	5	6	7	8
1. SBP	-	0.699**	0.159	0.091	0.097	-0.093	-0.073	0.018
2. DBP		-	-0.181	-0.201	-0.296	-0.408	-0.208	-0.046
3. SDNN			-	0.915**	0.735**	0.765**	0.688**	-0.044
4. RMS				-	0.565**	0.785**	0.576**	-0.199
5. LF					-	0.598**	0.653**	0.355*
6. HF						-	0.699**	-0.304
7. VLF							-	-0.018
8. LF/HF								-

Abbreviations: SBP, Systolic Blood Pressure. DBP, Diastolic Blood Pressure. SDNN, Standard Deviation of N to N beats. RMS, Root Mean Square. LF, Low Frequency. HF, High Frequency. VLF, Very Low Frequency. LF/HF, Low Frequency/High Frequency ratio.

*Correlation is significant at 0.05 (2-tailed)

**Correlation is significant at 0.01 (2-tailed)

Multiple regression was used to assess the effect of HRV variables (SDNN, HF, LF, VLF) on both SBP and DBP. With all predictor variables, SBP showed no significance R^2^ = 0.164, F (4, 28) = 1.370, *p* = .270. The standardized weights showed no variable as significant. Regression was not significant with DBP and predictor variables, R^2^ = 0.072, F (4, 28) = 2.419, *p* = .07. However, standardized weights in this model did show HF as significant (*p* = .019).

## Discussion

This study used a repeated measure design examining HRV and BP after paced breathing training followed by at-home self-training sessions for two weeks. Using HRV to understand the state of the ANS in real-time, along with demonstrating whether participants had parasympathetic or sympathetic dominance for either systolic or diastolic BP, provided valuable feedback. The visual data provided education to participants on the role of the ANS and its integration of body systems that control the body’s stress and recovering processes. [37].

In our sample of young adults with a family history of CVD, the results indicated an improvement in HRV from baseline to after HRV-BT training as demonstrated by an improvement in SDNN and TP. A similar study found that SDNN and PNN50 were lower in hypertensive patients, lending to the need to strengthen these time domains in this population. [38] When monitored over a 24-hour time period, SDNN values have been found to predict morbidity and mortality. [17] This demonstrates the effectiveness of the intervention in helping to improve the participant’s overall health. SDNN correlates with LF when paced breathing is synchronized with heart rate. In our sample this was exhibited when we compared SDNN and LF before and after the paced breathing. However, we found no significant improvement in SBP when comparing the means prior to and after training. Our findings did coincide with Lehrer and Gevirtz [36] in that the oscillations of the heart rate and breathing patterns became simple and sinusoidal during the HRV-BF training. The paced breathing training resulted in a decreased heart rate and higher SDNN, indicating a beneficial body response. It is well documented the influence of environmental stimuli on the ANS leading to dysfunctional CV control and HTN. [14, 39] In a younger adult population, it may be beneficial to teach paced breathing in order to increase HRV and decrease the possibility of morbidity in the future. As in our sample, there was a significant increase in SDNN.

In this study, we found a decrease in DBP while HRV increased. Mori and colleagues [40] also found an inverse relationship in HRV and DBP, but not SBP. In another study, researchers used mean arterial pressure and found that it correlated with HF. [41] However, both of these studies took place in an older population with comorbid factors. In a more recent epidemiologic study, the researchers found that DBP in those less than 50 years of age could be an added predictive factor of cardiovascular and mortality risks. [42] Our findings supported the relationship between DBP in this sample of young adults. We found a decrease in DBP with an increase in SDNN. Further, DBP showed an inverse relationship with HF, indicating that respiratory regulation was effective and parasympathetic dominance. It is noted that in younger, healthy individuals that HF decreases in the day and increases at night. [43] The sample for this study was young and healthy and may have had a lower HF at the beginning of the study since it took place during the day. Having young adults practice paced breathing during the day will assist in decreasing stress through enacting more parasympathetic activity of the ANS, lowering DBP and early CV morbidity.

When looking at multiple regression with all predictor variables in the model, there was no significance with SBP or DBP. However, HF did show some significance with DBP within this model. Most research relates SBP to long-term CVD effects. However, several studies have shown that DBP in younger aged persons may indicate a higher CVD morbidity. With this known and the association of DBP with HF and SDNN in this study, BF training for targeted DBP reduction could assist in slowing CVD development, particularly through stress reduction. There is minimal literature related to the specific effects of HRV on DBP. This is an area in need of further research.

Prevention through early interventions is crucial. Participants in our study indicated that anxiety and depression were both health concerns. Studies have found that there is a relationship between anxiety and hypertension [44] and depression and hypertension [45]. Teaching participants, with a family history of CVD, about an intervention that can assist in decreasing symptoms of anxiety and depression may help reduce the participant’s risk of early development of CVD in the future by maintaining the sympathetic and parasympathetic balance. Our study involved face-to-face sessions teaching paced breathing with immediate visual feedback followed by two weeks of at-home training using a mobile app. This design was well received as it has been reported that younger adults have a need for the immediacy of health information and technology that is readily available for use at their convenience. [37] Clinical implications include the need for providers to assess for family history of CVD in young adults and how much anxiety and stress the person is experiencing to start intervention early. Prevention efforts, such as paced breathing, at an early age could result in positive long-term outcomes.

Healthcare providers assessing young adults with a family history of CVD can provide these patients with options for reducing their risk of developing CVD. HRV biofeedback is a biobehavioral, nonpharmacological intervention that can be used by young adults with a family history of CVD to improve their HRV, which may assist in slowing the development of CVD. Additionally, paced breathing taught to participants in this study can be easily shared with patients in a clinical setting as a cost-effective intervention that patients can engage in at their convenience. Paced-breathing and HRV biofeedback training can be used to empower patients to take control of their cardiovascular health and used as a stress-reduction tool.

There were several limitations in this study including the sampling method and the sample size. As this was a pilot study, convenience sampling was used for data collection. However, this type of sampling method impeded our ability to draw inferences about the entire population. Furthermore, our sample was relatively homogenous, with primarily young females participating in the study, which could also be a result of convenience sampling. With only 34 participants, the small sample size may have decreased the statistical power of the results. There were notable trends observed in the data suggesting an effect with HF, SDNN and DBP. A larger sample could increase the statistical power and potentially a true impact on other variables. Our research team will focus on the continued enrollment of participants for reanalysis with a larger sample. Future research will use the same protocol with the addition of an anxiety and other well-being measures, as participants indicated that this is a health concern. For example, adding an anxiety assessment may help in determining the relationship between anxiety and HRV in individuals with a family history of CVD. Participants self-reported their medical history; however, no actual physiological data or pulmonary function testing were collected before the study. Undisclosed decreases in pulmonary function could skew results. Future studies should consider including pulmonary function testing before the study.

Increasing the amount and duration of the at-home paced breathing exercises can lead to a strengthening in vagal tone and potentially more substantial effects. Future studies could have participants practice at-home breathing exercises for longer periods. Additionally, contacting participants daily to discuss their progress may enhance compliance. In our study, the at-home treatment protocol was not supervised, and thus treatment adherence by participants was self-reported.

## Conclusions

With the current consideration of BP guidelines having the greatest impact on younger patients, careful consideration and future research should focus on those at-risk due to added nonmodifiable risk components, such as a positive family history of CV disease. Our study’s clinical implications demonstrate how the use of BF and paced breathing interventions could assist in early efforts to reduce early onset CVD. Health care providers can seek biofeedback practitioners to assist with training individuals or become knowledgeable about the various apps available and provide their own patient teaching. An individual cannot modify their genetic predisposition for developing CVD; however, this study supports the use of simple interventions such as paced breathing and BF training that can be introduced as a behavioral modification.

Teaching participants a simple, biobehavioral, self-management intervention that they can use at their convenience, empowers participants to embrace their health and practice exercises that can assist in decreasing the risk factors related to CVD. This allows for greater independence for young adults. Millennials and Generation Z may prefer self-directed healthcare and favor alternative holistic approaches. Paced breathing is merely one lifestyle modification that can be employed to mitigate their risk of developing CVD.

## Supplementary information

**Additional file 1**

## Data Availability

The datasets used and/or analyzed during the current study are available from the corresponding author on reasonable request.

## Abbreviations

Apps: Applications
ANS: Autonomic Nervous System
BF: Biofeedback
BP: Blood Pressure
bpm: Beats Per Minute
CNAP: Continuous Noninvasive Arterial Pressure
CV: Cardiovascular
CVD: Cardiovascular Disease
DBP: Diastolic Blood Pressure
HF: High Frequency
HR: Heart Rate
HRV: Heart Rate Variability
HRV-BT: Heart Rate Variability Biofeedback Training
HTN: Hypertension
IBP: Inter-Beat Interval
LF: Low Frequency
M: The statistical Mean value
mmHg: Millimeters of mercury
ms: milliseconds
ms2: Milliseconds squared
PNN50: The proportion of NN50 divided by the total number of NN (R-R) intervals.
RedCap: Research Electronic Data Capture
RMS: Root Mean Square
SBP: Systolic Blood Pressure
SDNN: Standard Deviation of N to N
SPRINT: Systolic Blood Pressure Intervention Trial
SPSS: Statistical Package for the Social Sciences
TP: Total Power
UMCIRB: University Medical Center Institutional Review Board
US: United States
USA: United States of America
VLF: Very Low Frequency
