# Supplementary material for: A pilot study investigating the relationship between heart rate variability and blood pressure in young adults at risk for cardiovascular disease
Source: Clin Hypertens. 2022 Jan 15;28:2. doi: 10.1186/s40885-021-00185-z (PMC8760819; doi:10.1186/s40885-021-00185-z)
Supplement: Supplementary file 1 — Additional file 1 [file 40885_2021_185_MOESM1_ESM.docx]

**At-Risk – Clinical HTN (8.23.21)**

| **Reviewer** | **Reviewer Comments** | **Response/How to Resolve?** |
| --- | --- | --- |
| **Editorial Office** | 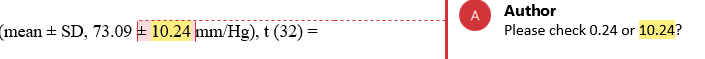 | 10.24 mm/Hg is correct  The last manuscript we submitted is correct. |
|  | 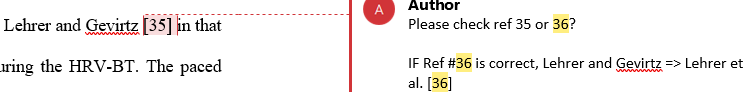 | 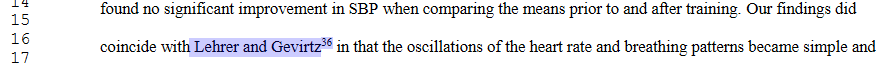  On the last manuscript we submitted (see above screen shot), we have it cited as reference #36 on page 11 which is correct and corresponds to the original reference page on that document. And the reference page reads as:  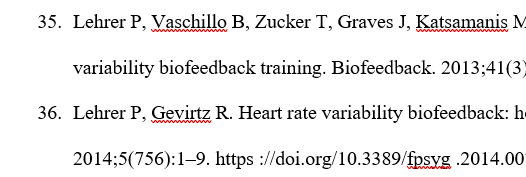  It appears on the edit request we received August 23, 2021 at 6:22 AM, somehow references 35 and 36 have been transposed from what was originally submitted.  The last manuscript we submitted is correct. |
|  |  |  |
